# Understanding the Utilization, Challenges, and Attitudes Towards Laboratory Testing and Radiological Imaging Among Chiropractors in Hong Kong: A Cross-Sectional Survey

**DOI:** 10.7759/cureus.40784

**Published:** 2023-06-22

**Authors:** Wai Ting Lee, Eric Chun-Pu Chu, Cherie Chau

**Affiliations:** 1 Research Committee, Chiropractic Doctors Assocaition of Hong Kong, Hong Kong, CHN; 2 Research Committee, Chiropractic Doctors Association of Hong Kong, Hong Kong, CHN

**Keywords:** hong kong chiropractor, chirpractic, laboratory testing, radiology, chiropractor

## Abstract

Objective

This cross-sectional survey aimed to investigate the utilization, challenges, and attitudes of chiropractors in Hong Kong towards laboratory testing and radiological imaging.

Method

An online survey was conducted between May 1, 2023, and June^ ^1, 2023. The target population for the survey was registered chiropractors in Hong Kong, which has a total of 325 practitioners. A total of 151 chiropractors participated in the survey, resulting in a response rate of 46.5%. The respondents provided information on their demographics, years of experience, practice settings, awareness of their right to request diagnostic tests, utilization of laboratory testing and imaging, and attitudes towards evidence-based practice. The survey data were analyzed using descriptive statistics.

Result

The survey findings revealed that a significant proportion of chiropractors in Hong Kong utilized laboratory testing and radiological imaging in their practice. Most respondents reported using laboratory testing to diagnose medical conditions and monitor existing conditions. Imaging is commonly used for diagnosing medical conditions and monitoring disease progression. However, a notable proportion of chiropractors reported facing rejection of their tests and imaging requests, which limited their ability to provide optimal care to their patients. The identified challenges included high rejection rates for specific imaging requests, leading to patient frustration, increased costs, and delayed diagnosis. Nevertheless, chiropractors in Hong Kong showed a strong belief in evidence-based practice and demonstrated a willingness to search for literature and incorporate it into their daily practice.

By addressing this issue, our aim was to gain valuable insights into the status of laboratory testing and radiological imaging among chiropractors in Hong Kong. The findings of this study underscore the necessity for collaborative efforts among chiropractors, healthcare professionals, healthcare facilities, policymakers, and insurance companies to effectively address the challenges identified and improve patient care outcomes. Specifically, enhancing access to diagnostic tests and working towards reducing rejection rates will play a pivotal role in supporting chiropractors' role as primary healthcare providers in Hong Kong.

## Introduction

Chiropractors are primary healthcare providers specializing in the diagnosis and management of neuromusculoskeletal complaints [1]. Over the past few decades, evidence-based practice (EBP) has greatly influenced chiropractic care [2], leading to the integration of crucial diagnostic tools such as laboratory testing and imaging. By utilizing these diagnostic methods, chiropractors can develop a comprehensive understanding of patient conditions, rule out major pathologies, enable accurate diagnoses, and create effective treatment plans.

In 1993, chiropractors in Hong Kong attained legal recognition and initiated the registration process under the Hong Kong Council [3]. As part of this registration, the chiropractic registration body in Hong Kong explicitly acknowledged the right of chiropractors to request diagnostic testing and imaging as primary care providers [4]. This regulation ensures high-quality chiropractic care in Hong Kong.

In recent years, chiropractors have become integral members of Hong Kong’s primary healthcare system. They provide effective chiropractic interventions and offer holistic education and counseling to the general public, positively affecting the overall health of the population [5].

Little is known about the use of diagnostic testing among chiropractors in Hong Kong. This study aimed to describe the utilization and attitudes towards laboratory and radiological testing among chiropractors in Hong Kong, as well as examine their attitudes toward EBP. By gaining insights into these aspects, we can further enhance the delivery of chiropractic care in this region.

## Materials and methods

Design

This study employed a cross-sectional survey design to investigate the utilization of laboratory testing, radiological imaging, and attitudes toward evidence-based practice (EBP) among chiropractors in Hong Kong. The study was approved by the ethics committee of the Chiropractic Doctors' Association of Hong Kong (Causeway Bay, Hong Kong; IRB ID: CDA20230605), and written consent was waived as consent was implied by the return of the completed survey. To ensure respondent anonymity, names, and other identifying information were not collected.

Setting

The target population of this study consisted of practicing registered chiropractors in Hong Kong. Both full-time and part-time chiropractors were recruited to participate in the survey.

Instrument

A comprehensive survey questionnaire was developed to gather data on the utilization of laboratory testing, radiological imaging, and attitudes towards evidence-based practice (EBP), drawing references from previous studies [3, 6]. The survey consisted of four sections: demographic data, laboratory testing, imaging, and EBP. The demographic data section included five questions regarding personal information and practice details. The laboratory testing and imaging sections consisted of 13 questions in total, exploring the utilization, purpose, and experiences of the requested tests and imaging. The EBP section comprised five questions, utilizing a 5-point Likert scale to assess the participants' attitudes toward EBP.

Data sources

The data for this study were obtained through the distribution of a survey using the Google Forms platform. The survey was distributed to all registered chiropractors in Hong Kong through their respective chiropractic associations. Official invitations to participate were sent via email, utilizing existing communication channels within the chiropractic associations. The completed survey forms served as the sole source of data for this study. The data were extracted from the survey responses and input into a spreadsheet format for further analysis.

Bias

To ensure the survey questions' clarity and estimate the time needed to complete the questionnaire, a pilot test was conducted with a small sample of chiropractors. All participants completed the survey within 10 minutes, and the pilot test revealed that the questions were clear and comprehensible. No adjustments were necessary.

Sample size

The sample size was determined using a rule-of-thumb estimation, indicating that each survey item required five respondents. Thus, 115 respondents were required for the study. This sample size was considered feasible given that 325 chiropractors were registered under the Hong Kong Chiropractors Council [7]. A survey conducted in Hong Kong collected 80 responses [3].

Analysis

The data extracted from the questionnaires were analyzed using Microsoft Excel. Frequency tables and descriptive statistics were employed to examine the current utilization of laboratory testing, radiological imaging, and attitudes toward EBP among chiropractors in Hong Kong.

## Results

The survey was administered online through a Google Forms link between May 1 and June 1, 2023. A total of 151 responses were received, resulting in a 100% completion rate.

Respondents

Table 1 presents the respondents’ demographic information. The participants were distributed across various age groups. The majority of the respondents (35.8%) belonged to the 20-29 age group, followed by the 30-39 age group, accounting for 23.8% of the participants. Age groups 40-49, 50-59, and 60+ years represented 20.5%, 11.9%, and 7.9% of the sample, respectively.

**Table 1:** Demographic information of the respondents

Characteristic		n=151 (%)
Age (years)		
	20–29	54 (35.8)
	30–39	36 (23.8)
	40–49	31 (20.5)
	50–59	18 (11.9)
	60+	12 (7.9)
Sex		
	Male	80 (53)
	Female	71 (47)
Years of experience		
	Less than 1 year	22 (14.6)
	1–5 years	41 (27.2)
	5–10 years	27 (17.9)
	More than 10 years	61 (40.4)
Type of practice		
	Group practice	82 (54.3)
	Solo practice	69 (45.7)
Acknowledgment of the right to request diagnostic imaging and laboratory examinations		
	Yes	144 (95.4)
	No	7 (4.6)

The study included a relatively balanced representation of both male and female participants. Males accounted for 53% of the respondents, whereas females accounted for 47%.

The participants exhibited varying levels of experience in chiropractic practice. The largest group consisted of respondents with more than 10 years of experience, comprising 40.4% of the sample. The next most prevalent group had one to five years of experience, accounting for 27.2% of participants. Respondents with five to ten years and one year of experience represented 17.9% and 14.6% of the samples, respectively.

The chiropractors in this study were engaged in different types of practice settings. The majority of participants (54.3%) were affiliated with group practices, while solo practitioners accounted for 45.7% of the respondents.

A significant majority of respondents (95.4%) reported awareness of their right to request diagnostic imaging and laboratory examinations, while only a small percentage (4.6%) reported being unaware of this right.

Laboratory investigation

A significant majority of the respondents (79.5%, n=135) reported requesting blood tests. Additionally, a high proportion of them (91.4%, n=138) expressed confidence in their interpretation of these results.

**Figure 1:**
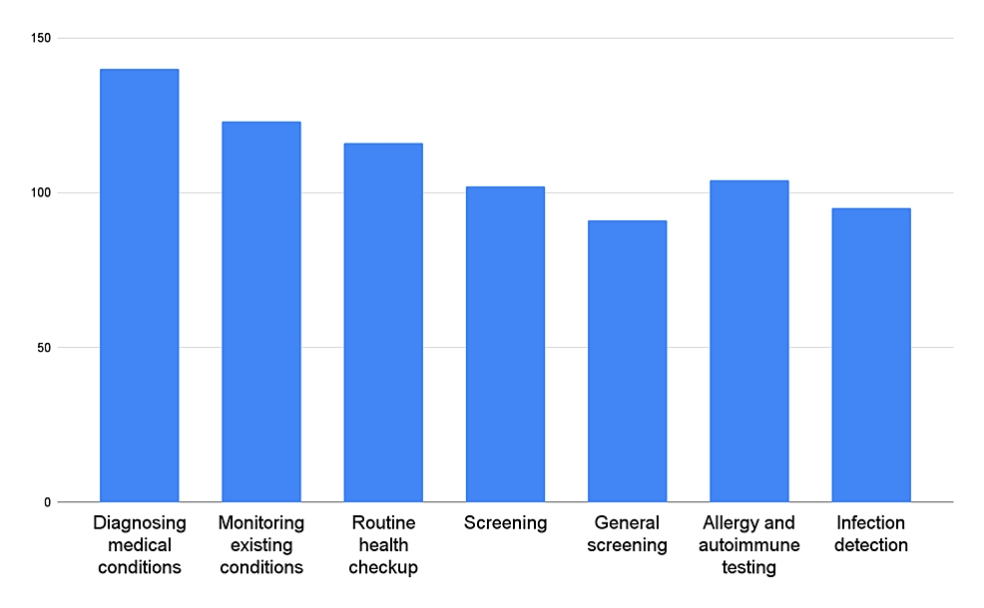
Purposes for the utilization of laboratory testing in chiropractic practice

Figure 1 presents the purpose of laboratory testing among the surveyed chiropractors.

The majority of respondents (92.7%) reported utilizing laboratory testing for diagnosing medical conditions. Furthermore, 81.5% of the participants reported utilizing laboratory testing to monitor their existing conditions, indicating its role in ongoing patient care. Laboratory testing was also frequently utilized for routine health checkups, as reported by 76.8% of respondents. Additionally, a significant proportion of chiropractors reported utilizing laboratory testing for screening purposes. This included both general screening (60.3%) and specific screenings, such as allergy and autoimmune testing (68.9%) and infection detection (62.9%). 

**Figure 2:**
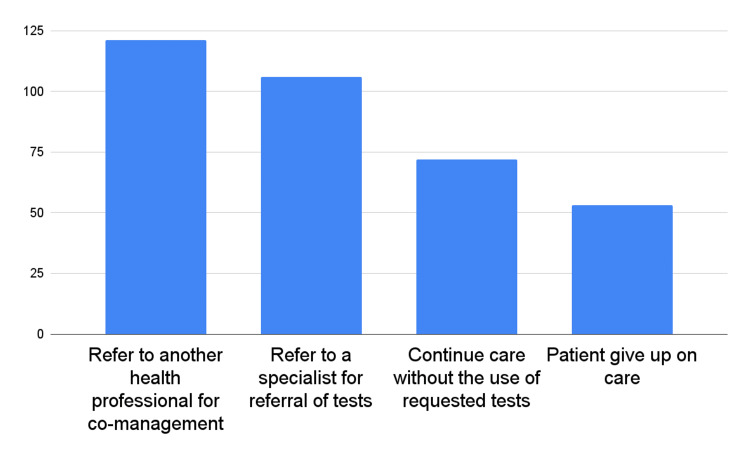
Situations experienced by chiropractors when blood test requests were rejected by medical laboratories

A noteworthy finding from this study was that a significant proportion of respondents (70.9%) reported that their blood test requests were rejected by medical laboratories. This study explored the situations experienced by chiropractors when test requests were rejected. The most common response by 81.2% of the respondents was to refer the patient to another health professional for co-management. Another common response by 71.1% of the respondents was to refer the patient to a specialist for a test referral, while 48.3% reported continuing care without undergoing the requested tests. Additionally, 35.6% of the respondents mentioned that patients gave up on care when their test requests were rejected. Figure 2 presents the situations experienced by chiropractors when their test requests were rejected. 

Imaging

All the surveyed chiropractors reported having requested imaging for their patients and expressed confidence in interpreting the results.

**Figure 3:**
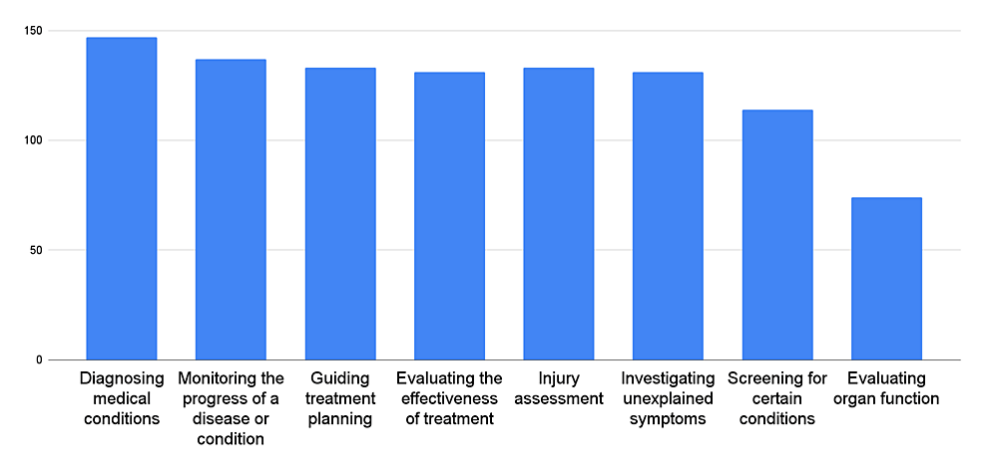
Utilization of imaging for chiropractic practice

The study results revealed the purposes for which chiropractors utilized imaging in their clinical practice. Figure 3 presents the utilization of imaging for chiropractic practice by the surveyed chiropractors.

These findings demonstrated the diverse applications of imaging in chiropractic practice. Most of the respondents (92.7%) reported utilizing imaging to diagnose medical conditions and monitor the progression of diseases or conditions (81.5%). Additionally, a substantial proportion of chiropractors utilized imaging to guide treatment planning (79.3%), evaluate the effectiveness of treatment (77.9%), assess injuries (79.3%), and investigate unexplained symptoms (77.9%). Screening for certain conditions (67.5%) and evaluation of organ function (43.9%) were also identified as the purposes for which imaging was performed.

A notable finding from this study was that a substantial proportion of respondents (80.1%) reported experiencing rejection of their imaging requests. The rejection rates of specific imaging requests were examined in detail. Radiography of the spine and the extremities revealed rejection rates of 23.3% and 82.9%, respectively.

MRI imaging requests also faced rejection, with MRIs of the spine and the extremities demonstrating a rejection rate of 31.8% and 86.8%, respectively. These findings indicate the presence of notable obstacles in obtaining MRI images for both spinal and extremity evaluations.

**Figure 4:**
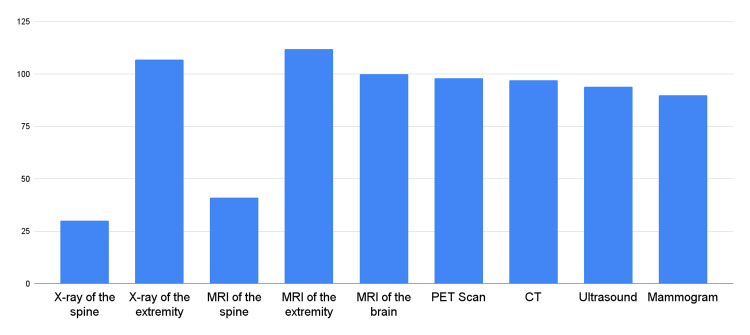
Type of imaging being rejected, as reported by respondents MRI: magnetic resonance imaging; CT: computed tomography; PET: positron emission tomography

Brain MRI demonstrated a rejection rate of 77.5%, indicating the challenges in obtaining brain MRI scans for chiropractic purposes. PET, CT, ultrasonography, and mammography techniques demonstrated a rejection rate of 76%, 75.2%, 72.9%, and 69.8%, respectively. Figure 4 illustrates the types of rejected imaging tests.

**Figure 5:**
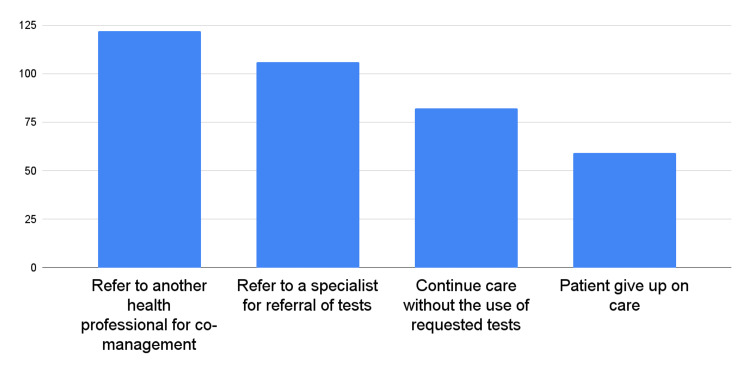
Situations experienced by chiropractors when imaging requests were rejected

This study demonstrated various situations when the test requests of the respondents were rejected by the imaging center. The findings indicate that a majority of chiropractors (83.6%) referred the patients to another health professional for co-management, whereas 72.6% referred the patients to a general practitioner for test referrals. Additionally, 56.2% continued care without the requested tests and 40.4% reported patients not returning for further care. Figure 5 presents the situations experienced by chiropractors when their imaging requests were rejected.

Practitioner attitude

All respondents expressed a strong belief in the importance of having access to medical laboratory investigations and radiological imaging in their practice. They recognized the value of these diagnostic tools in enhancing patient care and treatment outcomes. However, when their requests for tests were rejected, they expressed significant concerns about their ability to provide optimal care to their patients. The high prevalence of patient frustration (90.5%), increased costs for patients (87.8%), and delayed diagnosis (91.9%) were acknowledged as key concerns resulting from rejected test requests. Additionally, the respondents also expressed concerns regarding inadequate patient care (85.1%) and the exacerbation of the patients' symptoms (75%), due to the unavailability of the requested tests. Notably, a small proportion of respondents (6.8%) reported no effect from rejected test requests. However, 60.8% of respondents reported that the rejection of test requests might lead to third-party complaints from insurance companies.

Attitude towards evidence-based practice

Table 2 presents the attitudes of chiropractors towards evidence-based practice. Most of the respondents (90.1%) in this study expressed agreement regarding the use of EBP in their practice. Additionally, the respondents demonstrated a strong level of confidence in their ability to read and search for literature, with 89.5% expressing their preference for literature searches when faced with a clinical question.

A substantial number of respondents (81.5%) reported consistent incorporation of EBP into their daily chiropractic care practices. Furthermore, a significant proportion of respondents (78.5%) indicated their commitment to regularly updating their knowledge and skills regarding evidence-based chiropractic care practices.

**Table 2:** The attitude of chiropractors towards evidence-based practice

	All respondents N=151 (%)		
	Disagree or Strongly disagree	Neutral	Agree or Strongly agree
Evidence-based practice is useful in my practice.	7 (4.6)	8 (5.3)	136 (90.1)
If I have a clinical question, I prefer to search the literature for an answer.	7 (4.6)	9 (5.9)	135 (89.5)
I know how to read and search for the literature I need.	7 (4.6)	9 (5.9)	135 (89.5)
I always incorporate evidence-based practices into my daily chiropractic practice.	8 (5.3)	20 (13.2)	123 (81.5)
I regularly update my knowledge and skills regarding evidence-based practices in chiropractic care.	9 (5.9)	23 (15.2)	119 (78.8)

## Discussion

The present study emphasized the significance of diagnostic testing in the practice of chiropractors in Hong Kong, demonstrating their awareness of its importance in evaluating and addressing musculoskeletal complaints in patients [8]. By incorporating diagnostic testing, chiropractors can conduct comprehensive assessments that lead to more targeted and effective treatment strategies.

The survey findings indicate a high level of confidence among Hong Kong chiropractors in interpreting laboratory testing and radiological imaging results, reflecting their education and training that equip them with the necessary skills and knowledge to analyze diagnostic information [3, 9,10,11]. This proficiency enables chiropractors to make informed decisions regarding the appropriate treatment plans for their patients.

In practice, chiropractors encounter severe pathologies infrequently [12]. A specific survey conducted in Hong Kong revealed that malignancy is observed in approximately 0.25% of adults presenting with low back pain [13]. However, an important aspect of chiropractic practice involves the diagnosis of neurological [14, 15], gastrointestinal [16-18], and reproductive dysfunctions [19, 20]. Additionally, considering the possibility of inflammatory arthritis [21, 22], infections [23, 24], cardiovascular diseases [25-27], systematic disorders [28], fractures [28, 29], tendon ruptures [29, 30], and metastatic conditions [31,32] that masquerade as musculoskeletal disorders is crucial for providing chiropractic care. Therefore, having access to laboratory testing and radiological imaging is vital for chiropractors to conduct comprehensive examinations, ruling out any underlying severe pathologies and ultimately enhancing the quality of life for their patients [33].

Moreover, the study indicated that chiropractors utilize laboratory testing and radiological imaging for diagnosing and monitoring medical conditions and performing routine checkups and screenings, thereby underscoring their expanding role in preventive care [5,9].

With the integration of diagnostic testing into their practice, chiropractors are assuming the role of primary healthcare providers, contributing to the overall management of patients' health. This expanded role highlights the importance of adopting a multidisciplinary approach to healthcare and fostering collaboration between chiropractors and other healthcare professionals to deliver comprehensive and integrated care [5,10].

However, chiropractors in Hong Kong face notable challenges due to the high rejection rate of test requests. Consequently, chiropractors often refer patients to other healthcare professionals for testing or co-management purposes. This limitation significantly hampers their ability to provide optimal care, leading to delayed diagnosis, increased patient frustration, and the potential for inadequate treatment.

Laboratory testing and radiological imaging have become indispensable components of chiropractic practice. Addressing the barriers associated with communication and collaboration between chiropractors and medical laboratories or imaging centers is crucial to ensuring that they have the necessary tools to provide comprehensive and evidence-based care to their patients, ultimately improving overall healthcare outcomes. Overcoming these barriers is of paramount importance for optimizing the delivery of chiropractic services.

Nevertheless, the survey findings demonstrated a positive attitude towards EBP among chiropractors in Hong Kong. This positive attitude aligns with that of other healthcare professionals, such as medical doctors in Hong Kong and other countries [3, 17-20]. Most respondents recognized the value of EBP in their chiropractic care (90.1%) and expressed a willingness to consult the literature for answers to clinical questions (89.5%). This indicates a favorable inclination towards incorporating research evidence into the decision-making process.

Moreover, a significant proportion of the respondents (78.8%) reported regularly updating their knowledge and skills in EBPs, indicating their commitment to ongoing professional development and staying updated with the latest advancements in chiropractic care. These findings underscore the importance of providing continued support and resources to facilitate EBPs among chiropractors. By fostering an environment that promotes EBP, chiropractors can enhance the quality of care for patients and contribute to advancements in the field.

This study had some limitations. The online survey method and self-reported data would have introduced bias, and the study only adopted descriptive analysis, affecting the representativeness and generalizability of the findings. Also, this study did not explore the reasons behind test rejection or assess the level of knowledge regarding evidence-based practice among chiropractors. Therefore, further studies are required to address those limitations, explore the reasons behind test rejection, and assess the level of knowledge regarding EBP among chiropractors in Hong Kong.

## Conclusions

The present study provides valuable insights into the experiences, challenges, and attitudes of chiropractors in Hong Kong regarding medical laboratory investigations, radiological imaging, and EBP. These findings underscore the integration of laboratory testing and radiological imaging into chiropractic practice, indicating the potential for chiropractors to play a role as primary healthcare providers. However, the high rejection rate of test requests presents a significant obstacle for chiropractors in delivering comprehensive care to their patients. There is a need to improve access to diagnostic testing for chiropractors to optimize patient care. Collaboration between chiropractors and other health professionals is important for addressing barriers to comprehensive patient care. Furthermore, the positive attitude among chiropractors towards EBP provides a clear direction for enhancing the quality of care provided in this field; therefore, education on evidence-based practice should be integrated into chiropractic programs and continuing education.
